# Frailty, Cognitive Decline, Neurodegenerative Diseases and Nutrition Interventions

**DOI:** 10.3390/ijms20112842

**Published:** 2019-06-11

**Authors:** María Elena Gómez-Gómez, Sara C. Zapico

**Affiliations:** 1 CENAC Clinical Analysis Laboratories, Plaza Merced, 2, 33401 Avilés, Asturias 33401, Spain; Sealen2000@hotmail.com; 2 International Forensic Research Institute and Chemistry Department, Florida International University, 11200 SW 8 St., CP323, Miami, FL 33199, USA; 3 Anthropology Department, Smithsonian Institution, NMNH, MRC 112, 10th and Constitution Ave, NW, PO Box 37012, Washington, DC 20560, USA

**Keywords:** frailty, cognitive decline, nutrition, neurodegenerative diseases, Parkinson’s disease, Alzheimer’s Disease, free radicals, antioxidants

## Abstract

Currently the human population is aging faster. This leads to higher dependency rates and the transformation of health and social care to adapt to this aged population. Among the changes developed by this population is frailty. It is defined as a clinically detectable syndrome, related to the aging of multiple physiological systems, which prompts a situation of vulnerability. The etiology of frailty seems to be multifactorial and its pathophysiology is influenced by the interaction of numerous factors. Morley et al. propose four main mechanisms triggering the frailty: atherosclerosis, sarcopenia, cognitive deterioration and malnutrition, with their respective metabolic alterations. Malnutrition is associated with cognitive impairment or functional loss, but it is also known that an inadequate nutritional status predisposes to cognitive frailty. Additionally, nutritional factors that may influence vascular risk factors will potentially have an effect on dementia decline among patients with cognitive frailty. This review aims to describe the nutritional factors that have been researched so far which may lead to the development of frailty, and especially cognitive decline.

## 1. Introduction

### 1.1. Concept of Frailty

The fast increase of aging in the global population leads to the acquisition of aging-associated conditions. Recent reports pointed out that neurological disorders were the leading causes of DALYs (Disability-adjusted life-years), the sum of years of life lost (YLLs) and years lived with disability (YLDs) (276 million) and the second leading cause of death (90 million) [1]. Among them, frailty has attracted the attention of geriatric medicine in the recent years. 

Frailty is a clinical condition characterized by the greater vulnerability of an individual to stressors caused by deterioration in multiple physiological systems. Frailty is a syndrome that encompasses physical, social, and cognitive aspects and conceptually a condition of reversible pre-disability [2,3].

In an attempt to standardize a definition of frailty, Fried et al. [4] proposed a clinical phenotype of frailty, defining it as the situation of increased vulnerability for homeostatic resolution after a stressful event. This increases the risk of adverse outcomes, such as falls, disability, fractures, hospitalization, and mortality in elderly people living in the community. The original study is based on a secondary analysis of data obtained from a prospective cohort study (Cardiovascular Health Study) of 5210 men and women over 65 years of age [2,5].

This concept has been widely used, and focuses mainly on the physical dimensions. In detail, a set of five criteria are used to analyze the presence/absence of signs or symptoms (weight loss, exhaustion, sedentary behavior, slow gait, low muscular strength). Individuals with three or more of these five criteria have physical frailty, and those individuals with one or two criteria have pre-physical frailty [6,7].

In contrast, other definitions promote a multidimensional approach of frailty based on an evaluation according to “frailty indexes”, which are calculated considering the accumulation of possible deficits, such as the presence of diseases, abnormal laboratory values, signs and symptoms, or disabilities [8,9].

The etiology of frailty seems to be multifactorial and its pathophysiology is influenced by several factors. Morley proposes four main triggered mechanisms of frailty: atherosclerosis, cognitive deterioration, malnutrition and sarcopenia with associated metabolic disorders. Several cross-sectional studies have shown an association between frailty and alterations of biological markers, although these studies have not found a specific marker [10,11].

Dementia is also a common health problem in the elderly. Additionally, cognitive impairment is considered a component of frailty. The association between physical frailty and cognitive impairment or dementia has been investigated in a series of cross-sectional and longitudinal studies, finding a significant link between these two phenomena [12,13,14].

### 1.2. Concept of Cognitive Deterioration

As described above, epidemiological evidence suggests that physical frailty may be associated to cognitive impairment and decline in late life, incidence of Alzheimer’s disease (AD), mild cognitive impairment (MCI), vascular dementia (VaD), dementias with no AD, and AD pathology in older subjects with and without diagnosis of dementia [9]. Three Canadian studies correlated frailty with a cognitive decline over a period of 5 years and with dementia and AD at intervals of five and 10 years [9,15,16,17].

Recently, these epidemiological findings related to frailty and cognitive deterioration have prompted the International Consensus Group of the International Academy of Nutrition and Aging and the International Association of Gerontology and Geriatrics to propose the concept of “cognitive frailty” defined by the simultaneous presence of physical frailty and cognitive impairment (classification of clinical dementia [CDR] = 0.5) in elderly people without a clear diagnosis of dementia. It is noteworthy that cognitive frailty seems to be a precursor of neurodegenerative processes and AD [9]. Reversibility also characterizes cognitive frailty, making it a useful objective in terms of prevention. This could lead to a decrease of dependency in older people.

Currently, there is a dearth of epidemiological evidence for cognitive frailty, including its prevalence and impact [5]. The progression of cognitive frailty towards dementia is not currently clear. However, several longitudinal studies have investigated the possible association of this new and complex clinical phenotype with the adverse effects related to frailty, finding an increase of disability risk and all causes of mortality associated with some models of cognitive frailty [9,18,19,20].

### 1.3. Alzheimer’s and Parkinson’s Diseases

Alzheimer’s Disease (AD) and Parkinson’s Disease (PD) are the main neurodegenerative disorders (NDs) affecting the elderly. AD is characterized by a progressive loss of memory, language, and cognitive ability [21] while PD is characterized by severe motor symptoms including uncontrollable resting tremors, muscular rigidity, impaired postural reflexes, and bradykinesia [22]. Additionally, molecular mechanisms are different between these diseases. AD is characterized by the over-production of amyloid-beta peptide [21] while PD is marked by selective nigrostriatal dopaminergic degeneration and accumulation of alpha-synuclein protein in what are called “Lewy Bodies” [23]. However, in both diseases, the full molecular mechanism(s) leading to pathogenesis are unknown. It is suggested that classical aging mechanisms, such as oxidative stress and mitochondrial malfunction could play a role in the pathogenesis of these diseases and other associated neurodegenerative diseases like cognitive frailty.

### 1.4. Aging Brain

With aging there is an increase in cellular senescence of neurons and microglia, and as a result, an increase in apoptosis, aggregation of protein, mitochondrial disfunction with increased reactive oxygen species and oxidative damage to protein and lipids, and accumulation of DNA damage [24]. The brain is particularly vulnerable to oxidative damage, however it has low expression of antioxidant systems compared with other tissues, which results in the accumulation of neurotoxic peptides, such as amyloid-beta, promoting the development of AD [25]. Additionally, the brain needs high levels of energy, supplied by the anaerobic oxidation of glucose. With aging there is a decrease in neuronal glucose transporters, and as a result, a decrease in glucose uptake [26]. This is associated with a decline in cognitive function. Finally, there is a decrease in the functionality of the oxidative phosphorylation system (OXPHOS) in mitochondria, increasing reactive oxygen species (ROS), creating a vicious cycle of damage to the mitochondrial DNA and as a result, OXPHOS [27].

Finally, there is evidence suggesting that cognitive decline is associated with prominent neuroinflammation because the misfolded and aggregated proteins bind to microglia Toll-like receptors and CD4, triggering the inflammatory response [28]. Although this could protect the brain, if it is uncontrolled and persistent it could be destructive due to the release of ROS and reactive nitrogen species (RNS) [28].

## 2. Interventions

Currently, prescription drugs for AD only treat the symptoms, thus it is imperative to find a treatment to delay and/or prevent the onset of the disease. There is substantial epidemiological evidence to support the hypothesis that there are modifiable factors at the metabolic, vascular, and lifestyle levels, together with the late development of cognitive disorders [29]. Among them, the association between dietary habits and the development of AD seems promising [30].

For all models of frailty, cognitive and affective disorders, physical activity and nutritional status have been suggested as markers of frailty. In particular, cognition has been considered as the major component, also associated with adverse health outcomes. In this way, possible preventive interventions related to cognition, including AD and dementia, could prevent this syndrome and its associated components. Therefore, the prevention of frailty, nutritional deterioration and weight loss in fragile elderly people can be approached with nutritional intervention [9,14,31].

It is clear globally, how a suboptimal diet is a risk factor for non-communicable diseases (NCDs), in particular, recent studies have indicated that 11 million of deaths and 255 million DALYs were attributable to dietary risk factors, like high intake of sodium, low intake of whole grains, and low intake of fruits [32].

### 2.1. Impact of Nutrition, Sleep, and Exercise in Neurodegenerative Diseases

Malnutrition is associated with cognitive impairment or functional loss, but it is also known that an inadequate nutritional status predisposes to cognitive frailty. In aging, changes in body composition and cognitive frailty are clearly implicated in the syndromes of frailty and sarcopenia. One of the first questions, therefore, is whether it is possible to act on this cognitive frailty through nutrition and establish supplementation strategies at the population level or if the efforts should focus only on patients with specific deficiencies. Additionally, there are controversies regarding which diet or micronutrients could prevent the transition from cognitive frailty to dementia [28,33].

Another question arises when considering the eldest population (over 90 years old). Recent studies highlight that people over this age with physical disabilities and movement difficulties are more likely to suffer from dementia. Thus, these studies infer that physical decline induces cognitive decline and vice versa. As a result, current research is focused on the relationship between frailty, sarcopenia, cognitive frailty, and global functionality. The role of nutritional intervention is therefore of great importance in this whole network [34].The progression of the frailty syndrome has been associated with a more sedentary lifestyle, a decrease of the metabolic cell mass, and reduced energy use and energy intake. Lower dietary intake is also linked to the risk of a suboptimal nutritional status combined with micronutrient deficiency. Indeed, malnutrition is one of the possible causes of frailty, and this is clear in older people with malnutrition, who also have worse cognition [35].

Results published in the Journal of Alzheimer’s Disease in 2012, show that the existence of an adequate balance of the proportion of daily calories derived from carbohydrates, fats, and proteins can help maintain neuronal integrity and optimal cognitive function in the elderly [36]. In addition, both a high and low body mass index, lead to worse results in memory, orientation, attention, and calculation tests. The ideal is an average term with the body weight within a healthy body mass index [36].

Sleep is also pointed to as cause/effect of dementia and associated neurodegenerative diseases. In fact, observational studies support associations between disturbed sleep and cognitive decline. As a result, it could be a risk factor for neurodegenerative diseases, particularly AD [37]. Additionally, other studies have indicated that changes in the diet, particularly the adoption of a Mediterranean Diet (MeDi), could lead to a better sleep quality in older adults [38,39].

In contrast to these two previous parameters, the role of exercise in the prevention/improvement of cognitive decline is not clear. Some studies have indicated that exercise alone cannot protect against cognitive decline in older persons [40,41], while others have described how it does protect against AD [42].

### 2.2. Mediterranean Diet (MeDi) and Other Diets

A Mediterranean-style diet (MeDi) can help protect against dementia. It is based on the highest intake of vegetables, fruits, legumes, potatoes, whole grains, and nuts, in combination with a low-moderate consumption of fish, poultry, red meat, and wine. Olive oil is the main source of unsaturated fatty acids [9].

In several recent population studies, a Mediterranean diet was associated with slower cognitive decline, reduced risk of AD, transition between mild cognitive impairment (MCI) to AD, and decreased mortality in patients with AD [29,30]. In addition, the results of these population studies were confirmed by systematic reviews and meta-analyses showing that adherence to MeDi was related to a reduced risk of cognitive decline, MCI, AD, and progression from MCI to AD [43,44]. A six year study in Italy pointed out a decrease in the development of frailty in community-dwelling elderly men and women who adhered to a MeDi [45]. Additionally, the FINGER study found slowed cognitive decline in older persons using a combination of a MeDi, exercise, socialization, computer games and treatment of cardiovascular risk factors [41]. In a recent Randomized Control Trial (RCT) with 6.5 years of follow-up, nutritional intervention with an improved MeDi, including extra virgin olive oil or nuts seemed to improve global cognition [46]. This evidence confirms that a nutritional model based on the MeDi, characterized by a higher intake of monounsaturated fatty acids (MUFA), n-3 polyunsaturated fatty acids, fish, high levels of antioxidants from fruits and vegetables, and alcohol consumption at low to moderate levels can have a protective action against AD and dementia [29]. These studies attributed the effectiveness mostly to extra virgin olive oil. It improved memory and decreased oxidative damage in the SAMP8, a spontaneous animal model of AD [47]. Olive polyphenols also interfere with aggregation of amyloid-beta and produce epigenetic modifications [48].

Other considered diets, such as the Nordic diet were associated with preventive effects against cognitive decline. This diet is based on local food products of the Scandinavian countries and is characterized by a variety of berries, increasing polyphenols and antioxidants. Additionally, it is based on rye, oat, and barley as grain products. The source of unsaturated fatty acids is rapeseed [49].

In Asia, a diet characterized by a high consumption of fruits, nuts and seeds, tea, vegetables, whole grains, shellfish, milk and fish was inversely associated with the prevalence of frailty [50].

### 2.3. Role of Fruits and Vegetables in Prevention and Outcome of Cognitive Decline

In any of the aforementioned diets, fruits and vegetables seemed to play a key role in the prevention and outcome of cognitive decline. This is related to their characteristics: they are rich in vitamins, minerals, and antioxidants, low in energy density, and a source of dietary fiber [51,52]. These compounds can modulate different processes like detoxifying enzymes, supporting the immune system, modulating cholesterol synthesis, and acting as antioxidants [53].

Several studies have indicated that an increased intake of vegetables is associated with a lower risk of dementia and slower rates of cognitive decline in older age [51,52,54].

Other studies that analyzed the impact of nut consumption on cognitive function, demonstrated a better overall cognition at older ages [55]. Nuts contain MUFA and polyunsaturated fatty acids (PUFA). Nut intake decreased total cholesterol and LDL cholesterol and reduced the risk of cardiovascular disease and type 2 diabetes [56,57,58,59,60]. Since these factors have been related to cognition, this could be the rationale of improvement of cognitive function by nuts.

The studies related to the role of coffee consumption and risk for cognitive decline or dementia are inconclusive. However, recent meta-analyses conducted by Liu et al. [61,62] suggested that higher coffee consumption is associated with reduced risk for AD, although it recommends further randomized controlled trials or well-designed cohort studies.

Finally, several studies point to the positive effect of green tea in the outcome of cognitive function and reduced rates of neurodegenerative diseases [63,64,65].

### 2.4. Antioxidants: The Key to Decreasing Cognitive Decline?

As described above, the brain is very sensitive to oxidative damage caused by free radicals. Therefore, the reduction of oxidative stress and the protection of mitochondria could be an important objective in the prevention and treatment of dementia. This relationship has been investigated especially in the case of Alzheimer’s dementia [66].

Antioxidants interrupt the propagation of free radicals or inhibit the formation of free radicals by different mechanisms. Antioxidants help in scavenging the species that initiate the peroxidation, breaking the autoxidative chain reaction, quenching superoxide ions, and preventing the formation of peroxides [67]. In addition to the antioxidant enzymes produced by the body, low molecular weight antioxidants, such as minerals, vitamins, carotenoids, cofactors, glutathione, and polyphenols are crucial for antioxidative defense mechanisms of cells and organisms [68]. Ascorbic acid (vitamin C) and tocopherol (vitamin E) cannot be synthesized by a human [69]. In contrast, there are several molecules that are synthesized in the human body and possess an antioxidant effect including glutathione, lipoic acid, uric acid, taurine, keto acids, melatonin, coenzyme Q, and melanins.

#### 2.4.1. Flavonoids

Flavonoids are the most studied group of polyphenols. These are found in herbs, fruits, vegetables, and medicinal plants. They are well-known for their antioxidant, antimutagenic, anti-inflammatory, and anticarcinogenic properties [70], attributed to their antioxidant activities: scavenging of free radicals; suppressing generation of free radicals by inhibition of enzymes and chelating trace elements; and upregulating antioxidant defenses [68].

Many studies have revealed the beneficial role of flavonoids to the brain from citrus fruit [71,72,73], wine [71,74], green tea [75], and cocoa [75]. Their mechanism of action in the brain as described above is antioxidant, anti-inflammatory, anti-apoptotic, and signaling pathways modulatory agents through interactions with ERK and PI3K/Akt pathways [76]. Additionally, they increase cerebral brain blood flow, which could lead to an enhancement of cognition [77].

Some studies suggested the use of flavonoids as potential therapeutic agents for AD and PD [78], since it has been demonstrated that flavonoids slow down the development of AD-like pathophysiology and related neurodegenerative diseases by disrupting amyloid **β** protein production, activating α-secretase (ADAM10), and inhibiting β-secretase (BACE-1) [79].

Some studies have shown that olive oil reduces oxidative stress and the production of arachidonic acid in rat macrophages through the prostaglandin G/H synthase pathway. Subsequently, Moreno et al. have verified these previous findings and concluded that some minor components of olive oil, such as tyrosol and β-sitosterol, reduce the production of free radicals in macrophages. These results have helped to identify a possible therapeutic target for the management of the NDs. Morato et al. have shown that some phenolic compounds of olive oil (oleocanthals and triterpene acids) have strong neuroprotective effects and, therefore, can serve as a therapeutic intervention in the treatment of NDs. Additionally, they have reported that the Mediterranean diet, which is usually rich in olive oil, is associated with a lower incidence of NDs [80,81].

Other studies have shown that green tea prevents cytotoxicity due to its antioxidant properties. Later on, other works have confirmed that green tea prevents neurodegeneration in dopaminergic neurons. Mandel et al. have verified the free radical scavenger activity of green tea and its participation in several intracellular pathways, pointing out its therapeutic role in the treatment of NDs. Weinreb et al. have documented that the polyphenolic components of green tea, with a high antioxidant capacity, can function as neuroprotective agent and reduce the incidence of DNs. Following this line of research, it is suggested that the consumption of green tea can protect neurons from oxidative stress damage [80].

In 1973, Smiral et al. reported that diferuloylmethane, which is better known as curcumin, has anti-inflammatory activity. Later, the anti-inflammatory activity of curcumin as well as its potent antioxidant effect was corroborated in numerous pharmacological studies [82,83,84,85]. In fact, at high plasma concentrations, curcumin effectively scavenges hydroxyl and superoxide radicals. Some works have demonstrated that curcumin has neuroprotective effects against oxidative stress. Other studies have showed that curcumin has beneficial therapeutic effects in NDs, for example, in both PD and AD. Additionally, it has been reported that curcumin reduces oxidative damage in AD in a dose-dependent manner. Finally, Ishrat et al. have shown that curcumin has a protective role against cognitive deficits associated with AD. Therefore, it seems that curcumin has beneficial therapeutic effects in the NDs. Although it will be necessary to conduct clinical trials to unequivocally establish the therapeutic role of curcumin in the treatment of NDs [80].

#### 2.4.2. Carotenoids

Carotenoids are naturally occurring organic compounds produced by plants and algae, several bacteria, and fungi [86]. Apart from their antioxidant activity, carotenoids also facilitate the modulation of the cell cycle, apoptosis, cell differentiation [87], enhancement of the immune system, regulation of cell signaling pathways, promotion of growth factors, and adhesion molecules [88,89]. Carotenoids are highly lipophilic molecules that reside intracellularly to shield the membrane from oxidative stress [90].

The implication of carotenoids on the pathophysiology of AD and dementia has been widely studied [91]. Carotenoids delay disease progression by suppressing oxidative stress, decreasing amyloid **β** peptide production, and inhibiting pro-inflammatory cytokines [92].

Beta-carotenes (vitamin A) have potent antioxidant activity and, in particular, provide antioxidant protection in lipid-rich tissues, such as neurons. Thus, beta-carotene can neutralize lipid peroxidation related to oxidative stress. In addition, it has been shown that beta-carotene can protect neurons against hydrogen peroxide, one of the main contributors to neurotoxicity. It has been reported that a sufficient intake of beta-carotene in the diet reduces the incidence of AD and other related dementias. Furthermore, beta-carotenes improve mitochondrial function and recent studies have pointed out mitochondria as possible therapeutic targets for NDs. Considering these two factors, the use of beta-carotenes as protection against NDs is suggested [93].

The extract of saffron (*Crocus sativus*), crocin, can protect neurons against neurodegenerative processes, either by their anti-inflammatory or antioxidant effects. Some studies have demonstrated that crocin activates antioxidant enzymes and reduces oxidative stress in the hippocampus. In addition, other studies have demonstrated the antioxidant effects of crocin in rat brain tissue. Additionally, other works have shown the antioxidant effects of crocin against ND associated with memory impairment, such as AD. Moreover, additional studies have concluded that oxidative damage is closely related to neuronal defects and that crocin, as a potent antioxidant, can reduce cognitive deterioration due to cerebral ischemia, which is mediated by oxidative stress. Soeda et al. have reported that oxidative stress, which induces neuronal cell death, is reduced by crocin through preventing the activation of N-SMase (neutral sphingomyelinase), ceramide production, and phosphorylation of JNK (c-Jun kinase). Khalili et al. have demonstrated that crocin can reduce oxidative stress and, consequently neurodegeneration in an experimental animal model of AD. It seems that crocin, by enhancing the antioxidant defense system of the brain, reduces oxidative stress and prevents the development of NDs [94,95].

#### 2.4.3. Ascorbic Acid (Vitamin C)

Vitamin C is another well-known antioxidant, which has been considered the “gold standard” for expressing the antioxidant activity of plant-derived foods. In addition, it is involved in many other important functions such as the production of collagen, enzyme cofactor, iron absorption and stimulation of the immune system. Moreover, it has been proposed that vitamin C could be a neuromodulator of dopaminergic, glutamatergic and GABAergic and cholinergic neurons. Vitamin C is considered the most important water-soluble antioxidant in the extracellular fluid of the human body.

Research evidence has demonstrated the potential protective function of vitamin C in neurodegenerative diseases [96,97]. Some studies discovered the importance of vitamin C for cognitive performance. Other works introduced and described the benefits of vitamin C to protect neuronal cells against neurodegeneration. Additionally, Calabrese et al. (reviewed in [96]) have shown that ascorbic acid, by readjusting the redox state and deactivating heat shock proteins, can protect neurons against NDs. In addition, Fiona et al. (reviewed in [96]) have shown that vitamin C is crucial for the brain during an ischemic state and protects against NDs. Recently, it has been proposed that ascorbic acid could be beneficial for other types of neuropathies, for example, Charcot-Marie-Tooth disease type 1A [80,98].

#### 2.4.4. Vitamin E (α-tocopherol)

Vitamin E is a plant derived liposoluble compound which can interact with cell membranes as a free radical scavenger, intervening in chain reactions that produce free radicals [68].

Behl et al. suggested that α-tocopherol (vitamin E) and 17β-estradiol have neuroprotective effects against oxidative stress and can inhibit the progression of AD [99]. Some evidence suggests that tocopherols could play a role on neuroinflammation by regulation of Alzheimer-associated enzymes such as COX-2, 5-lipooxygenase (5-LOX), and NADPH oxidase [100], as well as the stimulation of phosphoprotein phosphatase 2A (PP2A), which plays a crucial role in tau homeostasis and it is lowered in AD brains [101]. Morris et al. have shown through a clinical study that high doses of vitamin E can reduce the risk of developing AD [102]. Some studies have suggested that a higher dietary intake of vitamin E may protect humans against the development of PD. Additionally, other works have demonstrated that treatment with vitamin E may slow the progression of AD. Grundman et al. (reviewed in [103]) have shown that vitamin E reduced functional impairment in patients with AD. Recently, Ulatowski et al. confirmed these findings and also emphasized the protective role of α-tocopherol in memory, learning, and emotional responses in the ND. Additional studies have documented that supplementation with vitamin E could reduce the incidence of AD. Savita et al. have presented additional evidence that α-tocotrienol (another active form of vitamin E) can block glutamate-induced neuronal cell death by suppressing early activation of c-Src kinase [103]. Finally, Bromley et al. have reported that vitamin E deficiency, or a mutation in its transporter, is associated with the progression of NDs [104].

#### 2.4.5. Vitamins B

B vitamins are essential and basic micronutrients that act as coenzymes necessary for the other basic nutrients to exert their action in the antioxidant-anti-inflammatory system.

Almost 50% of all apparently healthy elderly people have high blood levels of homocysteine, an amino acid that is formed from protein metabolism. Experimental results have shown that a high concentration of homocysteine in the blood destroys the blood cells that cover the inside of the blood vessels (endothelium), promoting the formation of thrombosis and increasing the level of LDL cholesterol, these factors lead to the development of atherosclerosis and cardiovascular diseases. Recent studies have indicated detrimental effects of high cholesterol for brain cells. It has also been shown that patients with vascular dementia and Alzheimer’s disease often have high homocysteine values, a risk factor for dementia. Several studies have reported that the selective administration of folic acid (vitamin B9), vitamin B6 and vitamin B12 reduces homocysteine levels and, therefore, the risk of circulatory problems in the brain [105,106].

Both epidemiological and clinical studies have shown that low levels of homocysteine and an adequate supply of vitamins B6, B9 and B12 are associated with a lower risk of developing Alzheimer’s dementia, and supplementation with B-complex vitamins can decrease the values of homocysteine and the risk of suffering from Alzheimer’s disease and its first symptoms. According to an observational study, high levels of homocysteine represent a risk factor that can predict the onset of Alzheimer’s disease at least seven years before the first clinical symptoms manifest. In addition, the increased level of vitamin B12 significantly reduced the risk of developing Alzheimer’s [105,106].

Cohort studies have so far provided conclusive evidence on the association between vitamin B deficiency and cognitive decline, additionally confirming that a high level of homocysteine is associated with poorer cognition. Randomized controlled trials have shown that supplementation with vitamins B can reduce homocysteine levels, although this is not translated into slower cognitive impairment, the improvement of cognitive function, or the reduction of the incidence of dementia. It is important to note that the most encouraging results have been reported in individuals with a higher level of basal homocysteine, suggesting that people with clear and defined deficiencies may be those who can most benefit from vitamin B supplementation [105,106].

#### 2.4.6. Vitamin D

Vitamin D receptors have been discovered in several key areas of the brain, increasing in neurons. On the other hand, it has been shown that there are enzymes in the brain that stimulate the local synthesis of calcitriol (the active form of vitamin D3). As a result of its multiple mechanisms of action, this vitamin could have an anti-inflammatory and protective effect on blood vessels and nerve cells, counteracting the development of dementia [107].

Vitamin D deficiency has been associated with frailty. In particular, data from the Third National Survey of Health and Nutrition has indicated that vitamin D deficiency is associated with prevailing frailty in both men and older women; participants with a serum vitamin D concentration <15 ng/mL had a 3.7 times greater chance of frailty. The connection between vitamin D and frailty can include two biological pathways related to muscle strength and bone health. Low levels of 25-hydroxyvitamin D [25 (OH) D] have a negative effect on calcium absorption and an increase of parathyroid hormone, leading to an increase in hip fractures. In addition, the binding of the activated form of 25-hydroxyvitamin D (1,25-dihydroxyvitamin D) to the receptors of skeletal muscle cells may contribute to protein synthesis and, therefore, a decrease in vitamin D levels could lead to a decrease in muscle strength. However, significant sex differences have been reported in relation to the association between vitamin D levels and frailty: older men, but not women, with inadequate vitamin D levels were more likely to be frail than men with enough vitamin D [108].

It has been demonstrated that vitamin D prevents cognitive deterioration in older rats with improvement of synaptic functions in the hippocampus [109]. In transgenic mice with AD, vitamin D supplementation decreased the level of beta-amyloid peptide and the number of amyloid plaques in the brain [110]. In several prospective studies, low levels of vitamin D in elderly patients have been associated with an increased risk of cognitive impairment [111,112,113]. Cross-sectional studies have also indicated that low circulating levels of vitamin D are associated with AD or AD with dementia [114,115,116].

Some forms of vitamins (e.g., vitamin E, ascorbic acid and beta-carotene) are well-known antioxidants. Although evidence has shown that they do not cross the blood-brain barrier (BBB) and cannot directly access the Central Nervous System (CNS) and associated neuronal structures, some reports have shown that they cross the BBB in small amounts and help stabilize and balance the oxidative microenvironment of the CNS [116].

#### 2.4.7. Organosulfur Compounds

These compounds are mainly present in vegetable species, like onion, garlic, and Chinese chive. They appear to combat oxidative stress associated age-related diseases, including neuroinflammation through their anti-apoptotic, antioxidant, and anti-inflammatory effect [117].

Several bibliographical references have demonstrated that garlic extract and its main ingredients have a powerful antioxidant activity. Chul et al. (reviewed in [118]) have recently reported that tiacremonone, isolated from garlic, inhibits the activation of p38 and protects against the deterioration of the substantia nigra. Brunetti et al. (reviewed in [118]) have shown that garlic supplementation can be effective against oxidative damage in the brain, although this effect was not observed in older animals. In addition, Mbyirukira et al. (reviewed in [118]) have demonstrated that garlic extract inhibits apoptosis of PC12 cells and improves brain function [118].

#### 2.4.8. Omega-3

Omega 3 fatty acids (**ω**-3) are unsaturated fatty acids, which contain a double bond at the third carbon end of the backbone. They are known to play an important role in various aspects of brain function, such as memory, cognitive function, synaptic transmission, and neuroplasticity [119].

The human brain is made up of 60% fatty acids, of which the omega-3 fatty acid, docosahexaenoic acid (DHA), accounts for between 20 and 30%. In cells of the brain and spinal cord (CNS), DHA is incorporated mainly in the phospholipids of plasma membranes and the membranes of cellular organelles (for example, mitochondria). DHA is especially abundant in the phospholipids of the nerve endings of the cerebral gray matter, where it plays a decisive role in the development, maintenance, and function of the central nervous system. Results of numerous experimental, epidemiological, and clinical studies have indicated that the type of fatty acids (omega-3 or omega-6) present in phospholipids depends, to a large extent, on the fatty acid composition of the diet [120].

Studies have shown that **ω**-3 has potent antioxidant effects. In addition, there are some studies that have suggested that **ω**-3 has neuroprotective effects against neurodegeneration induced by oxidative stress [121].

Moreover, a negative correlation has been reported between the levels of **ω**-3 in the blood (and other polyunsaturated fatty acids) and the risk of development of ND. Some studies have recognized that supplementation with **ω**-3 in elderly patients can reduce the incidence of AD. Other works have confirmed that continuous prophylactic use of an **ω**-3 supplement, as an antioxidant, may slow the progression of pathological disorders associated with oxidative damage, as seen in many NDs. Giselle et al. have shown that a diet enriched in **ω**-3 can decrease the production of beta-amyloid and its accumulation and, consequently, prevent its toxic effects. Recent studies have focused more on **ω**-3 supplementation and its effect on the risk of developing NDs [122,123].

Additional experimental (preclinical) research, as well as carefully controlled and monitored clinical trials, are necessary to determine whether ω-3 will be a useful and more traditional therapeutic complementary option for the treatment of NDs. The evidence from experimental studies on the beneficial effects of omega-3 PUFA supplementation is insufficient to recommend its implementation in the general population, either at the level of prevention or treatment for cognitive frailty or for established dementia. However, dietary recommendations to increase the amount of food intake with omega-3 PUFA and supplements for those that are deficient in these fatty acids, especially DHA, is indicated [124].

#### 2.4.9. Other Antioxidant Agents

In addition to the above agents, there are other compounds that function pharmacologically as antioxidants. Selenium, coenzyme Q10, melatonin, alpha-lipoic acid, 2.8. Szeto Schiller peptide-31, and estrogens are some of these other compounds [80]

Many of these agents can be administered as drugs (pharmaceutical formulations and devices) and modify the oxidative environment. In vivo, these agents may be useful to prevent early development, or at a minimum, help with the treatment of ongoing NDs [74,125].

## 3. Neurodegenerative Disorders and Gut Microbiota

Several lines of evidence suggest that the gut microbiota is an important part of the microbiota-gut-brain axis, which includes multiple bidirectional systems through which the gut microbiota and the brain communicate, encompassing hormonal (Hypothalamic Pituitary Axis, (HPA), neuronal (vagus nerve), and immune [126,127] systems. A healthy Gastro-intestinal tract in a homeostatic state has a normal and stable commensal intestinal microbiota and provides the host with nutrition and energy by producing vitamins [21]. Alterations associated with aging in the composition of the intestinal microbiota probably contribute to immunosenescence and the development of a proinflammatory phenotype (inflammatory aging). Inflammatory aging, in turn, can significantly alter brain function due to an increase in the expression of inflammatory cytokines and increase oxidative stress, the breakdown of the blood-brain barrier, the infiltration of peripheral immune cells, and glial cell activation. It is likely that these processes contribute to cognitive disorders related to aging and behavioral changes, such as anxiety, depression, and decreased sociability [128].

It was hypothesized that disease begins in the gut and spreads from the gut to the brain [21]. It has been shown that age-related changes in the composition of the microbiota play a role in the development of neurodegenerative disorders, including Parkinson’s disease (PD), affecting 1–2% of the population over 65 years of age. This disease is characterized by the accumulation of misfolded synuclein (Syn) that affects all aspects of the functioning of the bowel axis of the brain, including the central nervous, autonomic, and enteric systems [129]. Surprisingly, Syn aggregates, which are known to be substantially involved in the pathogenesis of PD, are found in the myenteric and submucosal plexuses of the enteric nervous system before they appear in the brain, indicating they can be spread from intestine to brain in a similar way to a prion [130]. The symptoms of constipation in patients with PD begin about ten years before the onset of motor function deterioration and these symptoms can be treated with antibiotics, thus, it is assumed that this disease could originate in the intestine [131]. Recent research with mice overexpressing Syn has indicated that gut microbiota is a key factor in motor deficits, activation of microglia, and Syn pathology [132]. In this study, supplementation with antibiotics improved the condition, while microbial recolonization promoted pathological conditions in adult animals, and oral administration of specific microbial metabolites to mice free of microorganisms promoted motor symptoms and neuroinflammation [128].

In numerous studies, a key role of the gut microbiota in the development of another neurodegenerative disorder, Alzheimer’s disease (AD) has been demonstrated. AD is a more common neurodegenerative disorder associated with cerebral accumulation of amyloid fibrils that drives neuroinflammation and neurodegeneration and results in a gradual loss of memory and severe deterioration of mental and behavioral functioning. It has been demonstrated that the bacterial species residing in the intestine release substantial amounts of amyloids and lipopolysaccharides, thus promoting the production of proinflammatory cytokines and modulating the signaling pathways involved in the pathogenesis of AD [133,134]. Numerous investigations have shown that AD can begin in the intestine and, therefore, is closely associated with imbalance of the microbiota. This suggests that modulation of the microbiome in vivo and, consequently, amyloidogenesis through specific dietary interventions and/or FMT (faecal microbiota transplantation) may provide a promising therapeutic strategy to prevent or delay the onset of dementia. Such approaches, however, have not been thoroughly investigated so far and could be the subject of future research [128].

## 4. Conclusions

**Figure 1:**
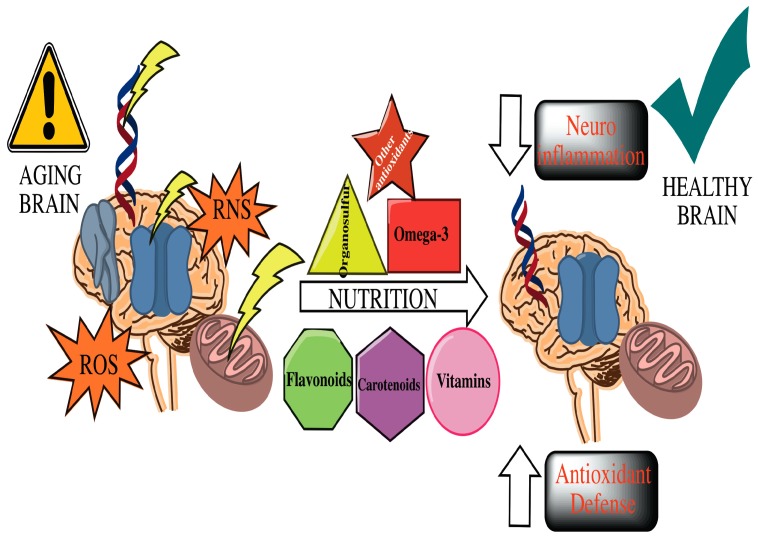
Summary of the effects of nutrition on aging brain and associated diseases.

One of the most important factors to consider in the development of cognitive deterioration is oxidative stress. Consequently, increasing antioxidants in the diet may be one of the therapeutic strategies in the management of these patients (Figure 1). There are numerous studies that have shown the effectiveness of antioxidants in the adjustment of oxidative stress, either by their function as free radicals scavengers, or potentiating the antioxidant effect. On the other hand, the inappropriate use of antioxidants could have side effects and toxicity at high doses. However, additional studies are required as well as clinical trials to increase the clinical evidence.
